# Reflections from PEARL for an era of systemic risks, uncertain futures and complex governance

**DOI:** 10.4102/jamba.v15i1.1485

**Published:** 2023-10-30

**Authors:** Arabella Fraser

**Affiliations:** 1 Department of Development Studies, Faculty of Arts and Social Sciences, Open University, Milton Keynes, United Kingdom

**Keywords:** disaster causation, systemic risks, governance, complexity, politics

## Abstract

Reflecting on the root cause framework devised for the research project preparing for extreme and rare events in coastal regions (PEARL) between 2014 and 2018 suggests a growing and evolving landscape of work building on the initial Pressure and Release (PAR) model. Empirical work using the PEARL framework added to debates about the nature, role and importance of institutions in the cross-scale interactions that drive disaster risk, even for small-scale disaster events. Going forward, its use opened up questions about our epistemologies and methodologies for root causes research in an era of systemic risk, uncertain futures and complex governance, and about tackling the layers of politics that can preclude action on reducing vulnerabilities.

**Contribution:**

The article suggests a new – and pluralised – agenda for root causes research going forward, which needs to be linked to critical understandings of the politics of root causes across disaster management and development actors and further action to reduce vulnerabilities.

From 2014 to 2018, through an EC-funded project called preparing for extreme and rare events in coastal regions (PEARL), a group of interdisciplinary UK and European researchers investigated how and why contemporary risks and vulnerabilities arose across four locations: Geneva, Italy; Hamburg, Germany; Rethymno, Crete, Greece; and St Maarten, Dutch Caribbean. All are coastal, urban locations differentially integrated into European structures and influence.^1^ The project was directly connected to the Integrated Research on Disaster Risk research programme on Forensic Investigation of Disasters (FORIN^2^), and PEARL research sought to further FORIN’s aims by expanding the range of case studies from which to draw systemic conclusions about disaster ‘root causes’. It drew on the conceptual indebtedness of the FORIN concept and framework to the original Pressure and Release (PAR) model, integrating hazard and vulnerability through investigation of both the physical and social drivers of risk, and their systemic origin. Like FORIN, however, we gave prime concern to the relevance of governance processes in mediating other causal factors (Oliver-Smith et al. 2016). The PEARL research specifically sought to address a long-standing gap in understanding the institutional pathways mediating broader mechanisms of PAR in relation to local drivers of risk, across contexts unevenly situated in relation to global and national power centres (Fraser et al. 2020).

In 2014, there were few other disaster ‘root cause’ frameworks or models to pull into our analysis. A root cause analysis framework developed by UNU-EHS on behalf of the German Committee for Disaster Reduction (DKKV 2012) made modifications to the FORIN framework that informed the PEARL approach (see Fraser, Paterson & Pelling 2016). The PEARL Framework disaster governance as a potential causal driver of risk mitigation or creation but also shifted our temporal perspective on root causes away from a solely historic lens to the analysis of how historic pathways to risk project into the present and future. Furthermore, the framework we devised for the project reframed FORIN (and the PAR model) for the empirical context at hand, namely the local governance of small-scale, but locally high-impact, disasters. This allowed us to test – and to some extent prove – the proposition that disaster risks, even at this scale and magnitude, manifest causal pathways that reach back to (but are not solely determined by) global-scale pressures. The 2008 global economic crisis, for example, initially exacerbated the pressure on resources available for disaster risk reduction in municipalities in Italy and Greece and worsened existing institutional fragmentation and the de-prioritisation of risk reduction. However, local institutional conditions also played a strong role in root causation. Anti-corruption drives in Italy prohibited pump-priming money on infrastructure from being spent on new flood defences in Genoa, while clientelistic relations in Greece prohibited more holistic forms of risk reduction.

By 2021, the PEARL framework and resulting analysis is supporting a growing, evolving and enriched academic landscape building critically on the early PAR model. The local context for PEARL and its derived framework is informing new, distinctive research exercises in urban-focussed risk root cause analysis, with increased emphasis also on multi-hazard risk interactions. This both reaches back to the need identified by the PAR model to deploy social vulnerability analysis as a domain for effective risk reduction and links to the growing need to understand the implications of contemporary decision-making for future urban development (Galasso et al. 2021).^3^ The Alliance for Resilient Urban SE Asia (ARUSEA) programme, a network funded by the UK Research Funding Agency to bring together social business leaders with disaster risk reduction academics and practitioners, also looks outside the role of the public sector to the role of private entrepreneurship as a possible domain for influence on ‘root causes’. This raises new questions not only about the structural constraints on vulnerabilities but also about the forms of emergent agency – public and private – that reconfigure them, and how to support multiple actors to address ‘root causes’.^4^

With the ongoing coronavirus disease 2019 (COVID-19) pandemic, a renewed context for disaster risk and climate change management has also emerged emphasising the need to understand risks as systemically interlinked (Pelling et al. 2021). The basic systems model of PAR had already been challenged to go further in explaining both the non-linearity and discontinuities of the interlocking ‘systems of systems’ that drive disaster occurrence (Zaidi 2018). From the holistic and open framework derived for PEARL, we aimed to establish a conceptual touch point that helps to frame this emerging agenda, avoiding some of the rigid social functionalism of systems thinking, but providing a more structured and policy-relevant framework than network thinking (Fraser et al. 2020). The PEARL analysis has subsequently been part of a lively debate about whether the application of network theory to disaster studies transcends the ‘search for root causes’ (McGowran & Donovan 2021). Both approaches raise important issues about the inextricable interlinkages between material and social worlds in risk creation processes, while the tension between the two approaches opens up necessary debates about the concepts we use to understand power, politics, temporality and space within these interactions. Renewed recognition within this debate that disaster ‘events’ are not discrete and one-off, but concatenate and cascade in ways that influence ongoing processes of risk accumulation, provides important insight for disaster causation studies going forward. This moves us forward from the original PEARL approach, which used bounded events as a heuristic device for interrogating causation.

Research in the PEARL project also positioned, but did not resolve, a debate of continued relevance about the methodological complexity of investigating root causes. From a pre-disaster perspective, this applied not only to social science research into disaster causation but also to the potential for greater interdisciplinary integration to support a systems science of disasters that could integrate subjectivity, power and culture (Abebe 2020). It raised questions that are still reverberating in disaster studies about the time frames at which it is necessary to understand root causes, how to connect this to present risks (especially if contemporary disasters are not yet manifest) and to possible futures (Duvat et al. 2021). From a post-disaster perspective, we extended the use of our root cause framework after the passage of Hurricane Irma in the Caribbean in 2017 to integrate root cause analysis into a loss and needs assessment (IHE-Delft 2018), thus modelling the potential for root cause analysis to also provide an effective baseline for transformational reconstruction and recovery.

Beyond questions about the science of root causes, the challenge of action on root causes remains urgent. Findings from the PEARL project – again, buttressed by the open, analytic frame and its application pre- and post-disaster – illustrate the layers of politics at play. The co-constitution of risks and vulnerabilities by local, national and global actors and the critical importance of informal institutional practices, of path dependencies and of spatial and temporal context do not make for easy policy blueprints. They call for new ways of governing flexibly and reflexively. Shifts in one policy domain may have unexpected consequences for risk reduction, demanding transparent deliberation of trade-offs and values (Scolobig 2017). This moves us on from thinking about the potential for structural change as just a question of organisational mainstreaming (although the answer undoubtedly lies beyond disaster risk management alone). In addition, although crises may allow a new or reinvented range of ideas and imaginaries to circulate, the idea that there is a singular ‘moment’ or ‘tipping point’ for progressive opportunity may need to be nuanced to allow for the positive and negative impulses and implications of different policymakers and policies to be assessed (Moatty, Grancher & Duvat 2021).

In the final stage of the PEARL project, post-Hurricane Irma, we were able to reflect on why and how different government constructions of knowledge and expertise emerged across political regimes in ways that opened up or closed down different arenas of possibility for action on root causes. In the Dutch and French Caribbean, territorial status (as an independent country or as French jurisdiction), government structure (in particular the type of devolution to local government) and colonial history (particularly administrative underdevelopment and the ongoing demands for local autonomy), all played their part. They promoted or constrained different forms of knowledge exchange and the de-prioritisation of certain issues such as land use regulation or migrant vulnerability. A further critical influence, however, was the role of multiple forms of political legitimation – within and between global, national and local government actors and audiences – in delimiting the possibilities for change (Borie & Fraser 2023). This led the French central government to push for a new climate change responsive agenda while the Dutch government kept tight control on recovery funds due to Dutch public concerns about financial misuse. The final outcomes of recovery planning, however, were mediated by their need to work with local governments, who were under their own pressures to legitimate fast economic recoveries and protect existing land use plans. The absence of community and citizens’ participation was notable (Borie & Fraser 2023; Collodi et al. 2021). Supporting accountability to and learning from and with vulnerable populations will be an imperative for action-oriented forensic research going forward, while moving away from automatic associations of FORIN investigations with blame and shame.

A future agenda for root causes research emerges, which is enmeshed in a more complex intellectual agenda as well as the ongoing need to advocate for social vulnerability in risk reduction. The contemporary environment for disaster studies prompts more diverse and critical engagements with questions of agency, subjectivity and knowledge than have hitherto been assimilated into ‘root causes research’, as far as it can or should be considered as a unified whole. A more plural research agenda must confront real-world tensions, however, of unfunded, fragmented mandates, in which the broader case for risk prevention is not yet won. We need to work not only from disaster risk management ‘out’ but also from development actors ‘in’, weighing up the politics of each at particular moments, and leveraging knowledge to understand the implications of a politics of business as usual, advancing incrementalism and promoting structural reform.

## Footnotes

1
http://www.pearl-fp7.eu/

2
http://old.irdrinternational.org/projects/forin/about-forin/

3
https://www.tomorrowscities.org/

4
https://www.arusea.org/
